# Genome Sequences of Five *Streptomyces* Bacteriophages Forming Cluster BG

**DOI:** 10.1128/genomeA.00502-17

**Published:** 2017-07-13

**Authors:** Richard Donegan-Quick, Zane A. Gibbs, Patricia O. Amaku, Joshua T. Bernal, Dana A. M. Boyd, Angela R. Burr, Rainna E. Coelho, Akpedje S. Dossou, Ryan M. Henry, Mai Huynh, Thalia A. Kanani-Hendijani, Guillermina Martinez, Tianeaka O. McClendon-Moss, Sergio Orozco, Johnny M. San Martin, Kaitlyn E. Stoddart, Madison M. Stringer, Rachel L. Villegas, Subhayu Nayek, Nikita Suri, Rebecca A. Garlena, Daniel A. Russell, Lee E. Hughes

**Affiliations:** a Department of Biological Sciences, University of North Texas, Denton, Texas, USA; b Department of Biological Sciences, University of Pittsburgh, Pittsburgh, Pennsylvania, USA

## Abstract

Cluster BG of the actinobacteriophage was formed upon discovery of five novel bacteriophages isolated by enrichment from their host, *Streptomyces griseus* subsp. *griseus* strain ATCC 10137. Four members of this cluster (BabyGotBac, Maih, TP1605, and YDN12) share over 89% average nucleotide identity, while the other (Xkcd426) has only 72% similarity to other cluster members.

## GENOME ANNOUNCEMENT

The genus *Streptomyces* is very diverse, with more than 576 validly published species names (1). Nevertheless, bacteriophages that infect *Streptomyces* have been described for only a few of these species (2–5). *Streptomyces* phages are currently grouped into 10 clusters (BA to BJ) within the actinobacteriophages based on nucleotide sequence similarity as well as their gene content (http://www.phagesdb.org) (6). In this work, five novel phages, which were used to established the cluster BG, were isolated using *Streptomyces griseus* subsp. *griseus* strain ATCC 10137 (*S. griseus*) as the host. To date, only *S. griseus* phages are found in the BG cluster.

All of the phages were isolated between 2012 and 2016 by enrichment of soils obtained from locations in Texas. Both BabyGotBac and Maih were isolated from soil samples collected in different locations in Denton, TX. Soil samples from Keller, TX, Valley View, TX, and Richardson, TX were used to obtain phages TP1604, Xkcd426, and YDN12, respectively. All of these phages exhibit a *Siphoviridae* morphotype and have a prolate capsid with a length-to-width ratio of approximately 1.6:1. The DNA of four of the phages, Maih, TP1604, Xkcd426, and YDN12, was sequenced using Ion Torrent at the University of North Texas, while the DNA of BabyGotBac was sequenced on an Illumina MiSeq at the Pittsburgh Bacteriophage Institute. Reads were assembled using Newbler and Consed software. Annotation utilized the DNA Master Software (http://cobamide2.bio.pitt.edu). All the cluster BG genomes were found to be circularly permuted. The genome lengths, G+C content percentage, number of open reading frames (ORFs), and GenBank nucleotide sequence accession numbers for each genome are given in Table 1.

**TABLE 1:** Five BG cluster phages

Phage	Yr of isolation	GenBank accession no.	Genome length (bp)	G+C (%)	No. of ORFs
BabyGotBac	2016	KY365739	57,165	69.2	72
Maih	2014	KU189325	57,256	69.3	70
TP1604	2012	KP876466	57,168	69.2	71
Xkcd426	2012	KU530220	64,477	68.8	78
YDN12	2012	KP876465	56,528	69.2	70

BabyGotBac, Maih, and TP1604 are the most similar to each other, with 99% average nucleotide identity (ANI). YDN12 has 89% ANI with those three phages. Most of the differences between YDN12 and the other three phages are seen in the right half of the genomes. Xkcd426 is the most different, with only a 72% ANI with the other four phages in the BG cluster. Xkcd426 is also over 7 kbp larger than the other members of the cluster. Much of the difference in genome size is due to an apparent insertion of more than 6 kbp beginning at approximately position 17800 through 24000. Five ORFs identified in this region have no homologous protein matches in the Phamerator database (7) and are thus designated as orphams. Nineteen additional orphams are found at various locations throughout the Xkcd426 genome.

### Accession number(s).

The whole-genome sequences have been deposited at GenBank under the accession numbers listed in Table 1.
